# emm typing and validation of provisional M types for group A streptococci.

**DOI:** 10.3201/eid0502.990209

**Published:** 1999

**Authors:** R Facklam, B Beall, A Efstratiou, V Fischetti, D Johnson, E Kaplan, P Kriz, M Lovgren, D Martin, B Schwartz, A Totolian, D Bessen, S Hollingshead, F Rubin, J Scott, G Tyrrell

**Affiliations:** Centers for Disease Control and Prevention, Atlanta, Georgia 30333, USA. rrf2@cdc.gov

## Abstract

This report discusses the following issues related to typing of group A streptococci (GAS): The development and use of the 5' emm variable region sequencing (emm typing) in relation to the existing serologic typing system; the designation of emm types in relation to M types; a system for validation of new emm types; criteria for validation of provisional M types to new M-types; a list of reference type cultures for each of the M-type or emm-type strains of GAS; the results of the first culture exchange program for a quality control testing system among the national and World Health Organization collaborating centers for streptococci; and dissemination of new approaches to typing of GAS to the international streptococcal community.


### *emm* Typing and Validation of Provisional M Types for Group A Streptococci[1]

**R. Facklam,* B. Beall,* A. Efstratiou,† V. Fischetti,‡ D. Johnson,§ E. Kaplan,§ P. Kriz,¶ M. Lovgren,# D. Martin,** B. Schwartz,* A. Totolian,†† D. Bessen,‡‡ S. Hollingshead,§§ F. Rubin,¶¶ J. Scott,## and G. Tyrrell¶**

*Centers for Disease Control and Prevention, Atlanta, Georgia, USA; †Central Public Health Laboratory, London, United Kingdom; ‡The Rockefeller University, New York, New York, USA; §University of Minnesota, Minneapolis, Minnesota, USA; ¶National Institute of Public Health, Prague, Czech Republic, #National Center for Streptococcus, Edmonton, Alberta, Canada; **Communicable Disease Group, ESR, Porirua, New Zealand; ††Institute of Experimental Medicine, St. Petersburg, Russia; ‡‡Yale University, New Haven, Connecticut, USA; §§University of Alabama, Birmingham, Alabama, USA; ¶¶National Institutes of Health, Bethesda, Maryland, USA; and ##Emory University, Atlanta, Georgia, USA

This report discusses the following issues related to typing of group A streptococci (GAS): The development and use of the 5' *emm* variable region sequencing (*emm* typing) in relation to the existing serologic typing system; the designation of *emm* types in relation to M types; a system for validation of new *emm* types; criteria for validation of provisional M types to new M-types; a list of reference type cultures for each of the M-type or *emm*-type strains of GAS; the results of the first culture exchange program for a quality control testing system among the national and World Health Organization collaborating centers for streptococci; and dissemination of new approaches to typing of GAS to the international streptococcal community.

The Lancefield M-typing system, a typical serologic system based on antigen-antibody reactions, is dependent on the preparation of type-specific antisera and extraction of a protein identified as M protein on the surface of group A streptococci (GAS) (1). The antisera against the M-protein antigens are produced with whole-cell streptococcal vaccines used to immunize rabbits. Acceptable antisera contain specific-precipitin antibodies and type-specific antibodies that must enhance the phagocytosis of the strain used to immunize the rabbit (2,3). The precipitin antibodies are made specific by absorption of the serum with streptococcal cells to remove the carbohydrate group antibodies and any cross-reactive precipitin antibody to heterologous M-type strains. Each rabbit antiserum is tested for reaction with antigens of all known M types.

Approximately half of GAS strains produce an apoproteinase, an enzyme that causes mammalian serum to increase in opacity. This reaction is called the serum opacity factor reaction, and the responsible enzyme is referred to as opacity factor (OF). The OF enzymes are OF type-specific because each M type that produces OF can induce type-specific OF antibodies that can be used in OF inhibition tests (3,4). Preparation of the OF antisera and specific details of the OF tests are described elsewhere (3,4). Some laboratories have used OF typing to predict M types in epidemiologic investigations. Even though it is not uniformly agreed that OF typing antisera results should be reported as M or provisional M-typing results, anti-OF antisera in many situations predict the M type in a type-specific manner. For reporting purposes, if

Address for correspondence: Richard R. Facklam, Centers for Disease Control and Prevention, 1600 Clifton Road, Mail Stop C02, Atlanta, GA 30333, USA; fax: 404-639-1379; e-mail: rrf2@cdc.gov.

[1]Information in this report was first presented at an International Workshop on Demonstration of *emm* Typing and Validation of Provisional M types for Group A Streptococci held at the Centers for Disease Control and Prevention, Atlanta, Georgia, August 26-28, 1997.

cultures are identified with M- or OF-typing antisera, they should be identified as M type or OF type.

Reference strains used to prepare the M antisera (several of which were originally described by Griffith in 1935) were historically obtained from the Lancefield collection, Rockefeller University, New York; however more recently, reference strains have been available from other reference laboratories. M-types 1 through 51 were designated in the laboratory of Dr. Rebecca Lancefield between 1928 and 1945. M-types 52 through 81 were submitted by various investigators to reference laboratories in Atlanta, London, New York, and Prague between 1965 and 1976 for confirmation. Some laboratories believe that certain strains in the Lancefield collection do not adequately express M protein and are unsuitable for the production of M-type antiserum. Preparing M-type antiserum or a typing system accurately related to the Lancefield system requires documented reference strains from one of the internationally recognized reference laboratories.[2]

In the Lancefield typing system, strains representing types 7, 16, 20, and 21 (originally described as Griffith) are not GAS but belong to groups C and G. M-type 10 is the same serotype as M12, M-type 24 is the same as M45, and M-type 35 is the same as M49; thus, designations of serotypes 7, 10, 16, 20, 21, 35, and 45 are not included in the Lancefield M-typing system 1 to 81 for GAS.

The *emm* gene of *S. pyogenes* is the gene that encodes the M protein. The M protein, responsible for the bacterium's capacity to resist phagocytosis, is a major virulence factor in GAS. The 5' ends of *emm* genes are highly heterogeneous and encode for the serotype specificity used for the M-typing system developed by Dr. Lancefield in 1928 (1). Producing type-specific M-typing antisera is difficult and specialized; no attempt has ever been made to produce them commercially, and only a few international reference laboratories prepare them. The resurgence of rheumatic fever cases in the United States and the emergence of severe infections (streptococcal toxic shock and necrotizing fasciitis) caused by GAS in the 1980s and 1990s indicated the need to reassess typing strategies for GAS. Since production of M-type precipitating antisera is very expensive and labor-intensive, the potential usefulness of a nonserologic typing system for GAS sequencing the 5' end of the M protein (*emm*) gene toward a molecular-based typing system was examined.

### *emm* Typing System for GAS

Before an *emm* genotype-based typing scheme was developed for GAS, the nucleotide sequence at the 5' ends of *emm* genes had been reported for many strains representing M-types 1-81 and several provisional M types (PT) (5,6). However, it was not always evident that true reference strains had been used. The knowledge gained from these studies provided the impetus for exploring the feasibility of an *emm*-based genotyping system. Subsequent studies based on sequencing the 5' *emm* genes from GAS reference strains and clinical culture specimens have now been published (7,8). In all studies involving *emm* sequence typing, 160 to 660 bases were sequenced from the 5' terminal end of the *emm* gene. The methods of *emm* sequencing and *emm* gene amplicon profiling restriction pattern techniques have been described (7,8). Two isolates are regarded as sharing the same *emm* sequence type if they are ≥ 95% identical over their 5' end 160 nucleotides (includes approximately 50 bp of the moderately conserved leader peptide-coding region), allowing for one frame shift or in-frame insertion/deletion of no more than seven codons (8). The results of *emm* gene sequencing of Dr. Lancefield's reference strains types 1 to 51 and reference strains of M-types 52 to 81, submitted to the Centers for Disease Control and Prevention (CDC) Streptococcus Reference Laboratory as potential new M types from 1967 to 1976 by various international investigators, are summarized below.

The 5' end *emm* sequences of the following CDC M-type reference strains matched the following sequences in GenBank that were submitted by other investigators: Types 1, 2, 3, 4, 5, 6, 8, 9, 11, 12, 14, 15, 17, 18, 19, 22, 23, 24, 25, 26, 27, 28, 29, 30, 31, 33, 36, 37, 39, 41, 43, 44, 46,

[2]Centers for Disease Control and Prevention, Atlanta, Georgia, USA; Central Public Health Laboratory, London, United Kingdom; The Rockefeller University, New York, New York, USA; University of Minnesota, Minneapolis, Minnesota, USA; National Institute of Public Health, Prague, Czech Republic, National Center for Streptococcus, Edmonton, Alberta, Canada; Communicable Disease Group, ESR, Porirua, New Zealand.

47, 48, 49, 50, 51, 52, 53, 54, 55, 56, 57, 58, 59, 60, 61, 62, 63, 64, 65, 66, 72, 73, 74, 75, 76, 77, 78, 80, 81. The accession numbers for these and many other *emm* sequences deposited in GenBank are listed in references 5-8 and at http://www.cdc.gov/ncidod/biotech/infotech_hp.html.

The 5' *emm* sequences from the CDC reference strains of GAS for the following M types were submitted to GenBank: M-types 13 (AF025950), 32 (L47325), 34 (L47324), 38/40 (L46817), 42 (L46799), 67 (AF025949), 68 (AF025948), 69 (AF035838), 70 (AF035838), 71 (L46652). The 5' *emm* sequences that were different from those submitted to GenBank by previous investigators (6) are M-types 13, 67, 68, and 79. For M-type 13, the *emm* sequence in GenBank was not from a recognized Lancefield typing strain; *emm* sequence AF025950 should be considered the *emm* sequence for Lancefield M-type 13. For M-types 67 and 68, the *emm* sequences in GenBank did not match those from CDC. The reasons for this are unknown; however, *emm* sequence AF025949 (M-type 67) and *emm* sequence AF025948 (M-type 68) were obtained from the reference strains submitted to CDC by the investigators who originally described these M types. For M-type 79, the *emm* sequence obtained by the CDC investigators matched the *emm* sequence in GenBank labeled M-type 80. Personal communication with the investigators who submitted the *emm* sequences for M-types 79 and 80 indicated a transcription error during submission of the sequences. Sequence U12004 is the correct sequence for M-type 79. The *emm* sequences from the CDC reference strains representing these four M types were confirmed by an independent laboratory.

CDC has additional data on *emm* typing of more than 1,500 GAS isolates from population-based studies, as well as random cultures from the United States and several other countries. Nearly 100% of the cultures could be genotyped by the *emm* typing system. In addition to determining the *emm* types of reference strains of M-types 1 to 81, several provisional type strains as well as new sequence type strains were typed by the *emm* typing procedure. Only one of more than 1,500 GAS isolates could not be *emm* typed by current methods. The *emm* sequence type of clinical isolates representing 35 distinct serotypes match the *emm* sequence type of the corresponding reference strain—included in this set of analyses are clinical isolates that underwent M or OF serotyping at one of the internationally recognized reference laboratories or elsewhere (8;9; B. Beall and D. Bessen, unpub. data).

The historical correlations between T type, OF reaction, and M type are largely unchanged when *emm* sequence type is substituted for M type (3,4,7,8). Because this observation is based upon the analysis of >3,000 clinical isolates, the T type and OF reactions together constitute an invaluable second-tier method for further confirmation of the grouping of closely related isolates, as well as for resolution of unrelated sets of organisms (R. Beall and R. Facklam, unpub. data).

When comparing a phenotypic-based typing scheme to a genotypic-based scheme, complete concordancy is not expected. Although for most types the M serotype is paired with a unique *emm* sequence type, there are several discrepancies. In five well-documented instances, 5' end *emm* sequences are >95% identical for two distinct M serotypes: M-types 27L and 77, 38 and 40, 44 and 61, 50 and 62, and 65 and 69 (10). The molecular basis for a lack of concordancy can differ for each unique M-type/*emm*-type pair. A few critical nucleotide substitutions at the 5' *emm* region could result in new, dominant antigenic epitopes that lead to the generation of a distinct serologic type. Alternatively, *emm* genes can occasionally undergo horizontal exchange and move onto a new genetic background, as appears to be the case for *emm*44 and *emm*61 (6); in this instance, additional dominant, polymorphic antigens may exist that can also be detected by M-typing sera. For the five examples cited above, introduction of a second typing scheme (T-agglutination and OF reaction) has proven useful in distinguishing between pairs displaying identical *emm* sequence types, but distinct M serotypes.

The discriminatory power of the genotypic *emm* sequence-typing scheme approximates that of the phenotypic M-serotyping scheme. For most M serotypes, there is a one-for-one relationship with a unique *emm* sequence type. The selection of 95% sequence identity as the cutoff value for defining the *emm* sequence type is based on empirical measures that best match the level of resolution achieved by M serotyping. In several examples, the *emm* sequence of one *emm*-type/M-type pair displays a relatively high level of sequence identity (but <95%) to a second, unique *emm*-type/M-type pair. For example, the *emm*3 and *emm*31 sequences share 91.3% identity over their first 160 bases; *emm*2 and *emm*73 are 89% identical over their first 160 bases of 5' end sequence, and this similarity is increased to 92.3% identity over their first 326 5' bases. Conceivably, certain genetic changes, such as a single bp insertion in the *emm* hypervariable region followed by a single bp deletion much farther downstream, could alter the reading frame of the gene and hence the antigenic structure and serotype of the *emm* gene product; however, this kind of variant has rarely been encountered in CDC surveys. Other genetic changes, such as synonymous substitutions, have no effect on phenotype. In some instances a single deletion or insertion of seven or fewer codons within the hypervariable 5' end 160 bp had no effect on the predicted M serotype (B. Beall, unpub. data). Therefore, an *emm* gene with less than 95% sequence identity to other *emm* genes may confer a new M serotype specificity. Thus, two isolates are regarded as sharing the same *emm* sequence type if they are $\geq$95% identical over their first 160 nucleotides, allowing for one frame shift or in-frame insertion/deletion of no more than seven codons (8). The 95% identity cutoff is not expected to match perfectly what can be achieved by serologic methods.

Most of the GAS isolates that are deemed nontypable by serologic methods can be genotyped through *emm* sequence determination. Furthermore, the M serotype does not always match the *emm* sequence-type (9,11). However, among the hundreds of strains analyzed by the streptococcal reference laboratory at CDC no discrepancies were observed between M serotype and *emm* sequence type. The full extent of putative M-serotype/*emm* sequence-type discordancies is not known, and the explanations for such a lack of congruency are numerous. A more complete understanding of the basis for discrepancies will be forthcoming as the *emm* sequence-typing method becomes more widely implemented.

The *emm*-typing system is a useful and reliable epidemiologic tool for subdividing GAS. Because it is independent of *emm* gene expression and can often discriminate between biologically distinct isolates that may be only weakly antigenic or nontypeable, *emm* sequence typing has the potential to classify isolates that have been difficult to type by serologic methods.

### Designation of M, Provisional M, and *emm* Sequence Types

GAS strains fall into three categories: Validated M types, provisional M types, and *emm* sequence types. If a laboratory has prepared antiserum to an unknown strain and the serum has type-specific precipitating antibodies, as well as bactericidal antibodies directed to that strain, verified by one of the six original reference laboratories, an M-type designation can be assigned to that strain. If two laboratories (at least one being one of the six reference laboratories) produces type-specific precipitating and bactericidal antiserum to the strain, the strain also qualifies as a new M type.

When a laboratory has prepared antiserum as described above to an unknown strain but the specificity of this antiserum has not yet been confirmed by a second reference laboratory, that strain is designated a provisional type. The requirements for conventional validation of new M types will be described elsewhere. A third category are sequence types or *emm* types, which are strains typed by sequencing the *emm* gene. If cultures are identified by *emm* sequencing, they should be reported as *emm* type.

### Validation Procedures and Nomenclature of New *emm* Sequence Type Strains

Published *emm* sequences from studies conducted in New Zealand and Australia included several new *emm* sequences included in GenBank (12,13). In addition, data from the CDC studies (B. Beall and R. Facklam, http://www.cdc.gov/ncidod/biotech/infotech_hp.html) indicated that more than 30 unknown *emm* sequences were identified among the 1,500 isolates of GAS that had been *emm* sequenced. A working group of representatives of each of the six international reference centers in Canada, New Zealand, Czech Republic, United Kingdom, and United States, was charged with establishing a definitive protocol both for submission of new *emm* sequences and for subsequent validation of new *emm* types. As an interim measure, unique 5' end *emm* sequences proposed as new *emm* types must be confirmed by a second laboratory; at least one of the two laboratories should be the streptococcal reference laboratory at CDC. If the uniqueness of the *emm* sequence can be confirmed by the second laboratory, the original investigator or one of the confirming laboratories will submit the findings to the Working Group, which will determine whether the strain should be assigned a new *emm* type number (e.g., *emm*94). In addition to sequence uniqueness, additional factors may be considered by the working group when making this decision; for example, previous requirements for assigning regular M- and provisional M-type numbers to strains were restricted to strains of particular clinical significance or to those occurring in a population "with significant frequency." Another remaining unresolved issue is whether or not all new *emm* reference strains should actively express M protein. A lack of surface expression of this *emm* gene product will preclude any possibility for correlation of *emm* type with classic serologic type or subsequent evaluation of biological significance. The relationship of *emm* sequence to biological function needs to be further explored.

CDC has validated six *emm* sequences submitted to GenBank by Australian investigators (12-14) and one *emm* sequence from a strain submitted to CDC by an investigator in the United States (15); these sequences should all be considered for official status as new *emm* types. Four additional isolates were examined at the CDC laboratory for which *emm* sequences had been submitted to GenBank (12-14). Strains STBSB75, ST1293, ST87/156, and STNS27 were shown to have the same *emm* types as M-type 70, M-type 76, PT2110, and PT5757, respectively. Therefore, these four *emm* sequences should not be accepted as new *emm* types, and their sequences should be reidentified in GenBank.

CDC has identified 10 new *emm* types from population-based studies of GAS invasive disease (7,8, unpub. data). In addition, eight new *emm* sequences from Brazil, six from Malaysia, three from Papua New Guinea, three from India, two from Ethiopia, two from Gambia, and one each from New Zealand and Chile have been confirmed by a second laboratory for *emm* sequence uniqueness, for 36 potentially new *emm* types.

### List of Reference Strains

The WHO Collaborating Laboratory for Reference and Research on Streptococci in Prague has prepared a database of the reference type strains to be used for research and antiserum production from information provided by the six international Reference Centers in Canada, New Zealand, Czech Republic, United Kingdom, and United States. Although all the reference strains on the list at one time had demonstrated survival in the in vitro bactericidal test (presumably reflecting functional M protein), the reference strains should be retested for survival in the bactericidal test before use in research. Dr. Lancefield's strains types 1 to 50 are listed at http://www.rockefeller.edu/vaf/

Because listing of type strains may be slightly different for each culture collection, when cultures are obtained, the strains should be properly identified. The strains for reference types 1 to 50 should be traced to the Lancefield collection, from which the M-typing system was derived. In the past, if a reference strain lost the capacity to express M protein on the bacterial cell surface, that strain was passaged in vivo and selected for the increased presence of M protein; therefore, many derivatives of the original reference cultures are in use by the reference laboratories, and none are known to have undergone change in their *emm* gene nucleotide sequence. The American Type Culture Collection (ATCC) has Lancefield's strains 1 to 50, the UK National Collection of Type Cultures has types 1 to 81, as well as the provisional types, and the Czech Culture Collection in Prague also has types 1 to 81 on deposit. Reference cultures 51 to 81 have been deposited in the ATCC by the CDC investigators who will also deposit the provisional type strains shortly. Plans are to continue the deposition of cultures of new *emm* types as they are confirmed.

Additionally, the CDC Streptococcus Laboratory has established an *emm*-type database at http://www.cdc.gov/ncidod/biotech/infotech_hp.html for use as a sequence comparison tool and additional documentation of all of the verified M and *emm* types. The database contains detailed information on the CDC method of *emm* typing and provides additional background on each reference strain and most known *emm* sequence types. Sequence comparisons can be done by using BLAST (Basic Local Alignment Search Tool). The data are prereviewed for errors and discrepancies, and all newly deposited *emm*-types are first verified directly by the CDC reference laboratory. At this time, until a complete validation protocol is established, investigators who discover new

*emm* sequence types should submit their isolates to the CDC Streptococcus Laboratory to confirm uniqueness. As a first step, investigators should search the CDC database for *emm* sequences; Genbank can be used as a second step, since it is always possible that neither database will necessarily have all known *emm* sequences of GAS at any given time. Furthermore, the CDC database will include the accepted *emm* type designation for those types, whereas a possible incorrect *emm* sequence may have been submitted to GenBank by an investigator.

### Validation of Provisional M Types to New M and *emm* Types

The Table lists the provisional M-type strains and the status of validation. Collaborative investigations involving the six reference laboratories included several other provisional M-type strains and has confirmed the following: PT179 fulfills all the phenotypic criteria for an M type; however, only anti-OF serum has been prepared. Therefore, the status of this strain as a potential new M type remains on hold until further supporting data can be provided. PT4854, Colindale Laboratory, United Kingdom (UK), had a closely matching *emm* type as M-type 43; this finding correlated to serologic tests at CDC and UK laboratories as M-type 43 that demonstrate that both strains reacted with M43 typing antiserum. PT3800, Prague, Czech Republic (CZ), has the same *emm* type as M-type 65, which correlated to serologic tests performed at the National Streptococcus Laboratory in Edmonton, Canada, showing that both strains reacted with M65 typing antiserum. PTYE327 (CZ) has the same *emm* type as PT2841(UK), which correlates to serologic tests performed in both the Colindale and Prague laboratories. PT1437, Porirua Laboratory, New Zealand (NZ), has the same *emm* type as PT4245 (UK), which correlates to serologic tests performed in both the UK and NZ laboratories. ST2974.95 (CDC) has the same *emm* type as PT5118 (NZ). ST2974.95 was shown to be PT5118 in serologic tests performed in the Porirua Laboratory. In summary, PT4854, PT3800, PTYE327, PT1437, and ST2974.95 should be identified as *emm*43 (M43), *emm*65 (M65), *emm*87 (M87), *emm*89 (M89), and *emm*92, (M92) respectively. The sources of most provisional types were first documented in 1985 (16).

The following *emm* types were isolated from patients with severe invasive disease in the United States; *emm*82, *emm*83, *emm*86, *emm*87, *emm*88, *emm*89, and *emm*92. These isolates comprised 12.7% of all isolates identified during these studies. Types *emm*82, *emm*85, *emm*87, and *emm*89 were associated with epidemic investigations in the United States. Many of these new *emm* types were also identified from other countries, including Argentina, Brazil, Bulgaria, Chile, Colombia, Denmark, India, Korea, Malaysia, New Guinea, and Poland. Other reference laboratories have reported these new M types (by using provisional type identifiers) in a variety of infections, including rheumatic fever and acute glomerulonephritis (17,18).

Table. Recommended action for M and *emm* designation for provisional M types[a]

| Provisional type | ID number | CDC ID number | New *emm* or M type |
|---|---|---|---|
| PT180(UK)[b] | NCTC12062 | SS-1395 | 82 |
| PT2110(UK) | NCTC12064 | SS-1400 | 83 |
| PT2233(UK) | R75/2233 | SS-1449 | 84 |
| PT2612(UK) | R76/2612 | SS-1447 | 85 |
| PT2631(UK) | R76/2631 | SS-1448 | 86 |
| PT2841(UK) | NCTC12065 | SS-1399 | 87 |
| PT3875(UK) | R67/3875 | SS-1455 | 88 |
| PT4245(UK) | NCTC12067 | SS-1397 | 89 |
| PT4931(UK) | NCTC12068 | SS-1396 | 90 |
| PT5757(UK) | NCTC12056 | SS-1398 | 91 |
| PT5118(NZ) | | SS-1460 | 92 |
| PTPotter41(UK) | R76/2631 | SS-1493 | 93 |

[a]For definitions of M and *emm* designations, see section in text, Designation of M, Provisional M, and *emm* Sequence Types.
[b]Abbreviations; UK= United Kingdom, NZ= New Zealand.

### An External Quality Assurance Typing Program among International Reference Laboratories

At the request of the World Health Organization (19), a quality assurance program on GAS typing has been established by the Central Public Health Laboratory, London, United Kingdom. Ten GAS isolates were examined by six different laboratories, and the results of the first distribution showed a very good correlation between laboratories. *Emm* typing by one laboratory correlated very well with M typing by the other five laboratories. Only minor differences were noted among the T- and OF-typing results; no errors were found among the M-typing results. Responsibility for sending 10 cultures to the other five centers twice a year would be rotated among the six reference centers and a report of the quality assurance program will be presented at the next International Lancefield Society meeting in New Zealand in the fall of 1999.

Dr. Richard Facklam is chief of the Streptococcus Laboratory, Division of Bacterial and Mycotic Diseases Division, National Center for Infectious Diseases, CDC. His major fields of interest include improvement in laboratory procedures for the diagnosis of acute respiratory tract infections, taxonomy of streptococci and related gram-positive cocci, identification of virulence factors associated with bacterial respiratory pathogens, and development of new systems for epidemiologic study of the transmission of bacterial respiratory pathogens.

### References

1. Lancefield RC. The antigenic complex of *Streptococcus haemolyticus*. I. Demonstration of a type-specific substance in extracts of *Streptococcus haemolyticus*. J Exp Med 1928;47:91-103.
2. Lancefield RC. Differentiation of group A streptococci with a common R antigen into three serological types, with special reference to the bactericidal test. J Exp Med 1957;106:525-44.
3. Johnson DR, Sramek J, Kaplan EL, Bicova R, Havlicek J, Havlickova H, et al. Laboratory diagnosis of group A streptococcal infections. Geneva: World Health Organization; 1996.
4. Widdowson JP, Maxted WR, Grant DL, Pinney AM. The relationship between M-antigen and opacity factor in group A streptococci. Journal of Genetic Microbiology 1971;65:69-80.
5. Kaufhold AA, Podbielski A, Blokpoel M, Schouls L. Rapid typing of group A streptococci by use of DNA amplification and non-radioactive allele-specific oligonucleotide probes. FEMS Microbiol Lett 1994;119:19-26.
6. Whatmore AM, Kapur V, Sullivan DJ, Musser JM, Kehoe MA. Non-congruent relationships between variation in *emm* gene sequences and the population genetic structure of group A streptococci. Mol Microbiol 1994;14:619-631.
7. Beall BB, Facklam R, Thompson T. Sequencing *emm*-specific PCR products for routine and accurate typing of group A streptococci. J Clin Microbiol 1996;34:953-8.
8. Beall BB, Facklam R, Hoenes T, Schwartz B. Survey of *emm* gene sequences and T-antigen types from systemic *Streptococcus pyogenes* infection isolates collected in San Francisco, California; Atlanta, Georgia; and Connecticut in 1994 and 1995. J Clin Microbiol 1997;35:1231-5.
9. Bessen DE, Izzo MW, Fiorentino TR, Caringal RM, Hollingshead SK, Beall B. Genetic linkage of exotoxin alleles and *emm* gene markers for tissue tropism in group A streptococci. J Infect Dis. In press, 1999.
10. Facklam R, Beall B. Anomalies in *emm* typing of group A streptococci. Adv Exp Med Biol 1997;418:335-7.
11. Desai M, Tanna A, Efstratiou A, George R, Clewley J, Stanley J. Extensive genetic diversity among clinical isolates of *Streptococcus pyogenes* serotype M5. Microbiology 1998;144:629-37.
12. Relf WA, Sriprakash KS. Limited repertoire of the C-terminal region of the M protein in *Streptococcus pyogenes*. FEMS Microbiol Lett 1990;71:345-50.
13. Relf WA, Martin DR, Sirprakash KS. Identification of sequence types among the M-nontypeable group A streptococci. J Clin Microbiol 1992;30:3190-4.
14. Relf WA, Martin DR, Sriprakash KS. Antigenic diversity within a family of M proteins from group A streptococci; evidence for the role of frameshift and compensatory mutations. Gene 1994;144:25-30.
15. Boyle MDP, Hawlitzky J, Raeder R, Podbielski A. Analysis of genes encoding two unique type IIa immunoglobulin G-binding proteins expressed by a single group A streptococcal isolate. Infect Immun 1994;62:1336-47.
16. Fraser CAM, Colman G. Some provisional M-types among *Streptococcus pyogenes*. (Lancefield group A). In: Kimura S, Kotami, Shiokaswa Y, editors. Recent advances in streptococci and streptococcal diseases. Proceedings IX Lancefield symposium on streptococci and streptococcal diseases. Berkshire (UK): Reedbooks; 1985. p. 35-6.
17. Martin DR, Voss LM, Walker SJ, Lennon D. Acute rheumatic fever in Auckland, New Zealand: spectrum of associated group A streptococci different from expected. Pediatr Infect Dis J 1994;13:264-9.
18. Colman G, Tanna A, Efstratiou A, Gaworzewska ET. The serotypes of *Streptococcus pyogenes* present in Britain during 1980-1990 and their association with disease. J Med Microbiol 1993;39:165-78.
19. World Health Organization. Report of an informal consultation of directors of WHO Collaborating Centres on Streptococci; 1994. Report No.: WH0/CDS/BVI/96.6.
